# Collaboration With Behavioral Health Care Facilities to Implement Systemwide Tobacco Control Policies — California, 2012

**DOI:** 10.5888/pcd12.140350

**Published:** 2015-02-05

**Authors:** Lauren Gordon, Mary V. Modayil, Jim Pavlik, Chad D. Morris

**Affiliations:** Author Affiliations: Lauren Gordon, California Department of Health Care Services, Sacramento, California; Mary V. Modayil, Institute for Population Health Improvement, University of California–Davis Medical Center, Sacramento, California; Jim Pavlik, University of Colorado, Anschutz Medical Campus Department of Psychiatry, Aurora, Colorado.

## Abstract

The California Tobacco Control Program (CTCP) administered 4 regional trainings in 2012 to staffers at CTCP-funded projects, tobacco control coalitions, several county departments of mental health and alcohol and drug, and administrators and providers from behavioral health care facilities. These trainings focused on the special tobacco use cessation needs and opportunities for cessation among persons with mental illness or substance abuse disorders, and they provided information about cessation and smoke-free policies. CTCP surveyed county and private behavioral health care programs to assess their readiness for adopting tobacco control strategies at treatment facilities. Between baseline and follow-up we found a decrease in the proportion of organizations at the precontemplation or contemplation stages of change and twice as many organizations at the action and maintenance stages of change. Significant obstacles remain to implementing policy: many agencies have concerns about going tobacco-free. But significant progress has been made, as evidenced by new policies and a growing number of tobacco-free coalitions consisting of public health agencies, behavioral health care agencies, and local hospitals.

## Objective

In California, 27.7% of people who experienced serious psychological distress in the last year (2011–2012) reported smoking, compared with 12.6% of the general population (1). To address the needs of this at-risk population, the California Tobacco Control Program (CTCP) surveyed county and private behavioral health care programs to assess their readiness for adopting tobacco control strategies at behavioral health treatment facilities. Themes that emerged from surveys of key informants guided 2012 regional behavioral health care trainings. Training provided evidence-based guidelines, and participating agencies created rapid improvement plans for implementing tobacco control strategies.

## Methods

In 2011, CTCP conducted key informant interviews by telephone with 17 CTCP grantees, all of whom ran county or private behavioral health care programs. We selected key informants who reported plans to use state tobacco control funds to address the cessation needs of people with mental illnesses or addictions. Qualitative data were obtained on current practices to address nicotine dependence, established treatment relationships, current policies, expected challenges, needed educational materials, and buy-in from local decision makers regarding implementation of tobacco-free policy and systems change. Additionally, we asked key informants whether they were aware that Mental Health Services Act grant funds were available through the California Mental Health Services Act for innovative cessation projects for people in need of behavioral health treatment. A brief 9-item interview, which included open-ended items and in-depth probes, was created with input from tobacco control and behavioral health county partners. For analysis, CTCP used an inductive qualitative approach to code emergent interview themes.

Subsequently, in 2012, we chose 4 locations from the 17 originally surveyed, and regional trainings were conducted by the University of Colorado Behavioral Health and Wellness Program (BHWP). Participating community agencies in Sonoma, Shasta, Santa Cruz, and San Diego were selected on the basis of local decision makers’ buy-in for implementing systems change and tobacco-free policy strategies to support people in need of the behavioral health treatment. Each training session had an average of 50 participants with the exception of the training at Sonoma County, which received special permission to have 100 participants. During trainings, participants learned evidence-based strategies for integrating tobacco cessation and tobacco-free policies into daily behavioral health treatment. Participants further engaged in strategic planning and developed rapid improvement plans by using an established plan-do-study-act (PDSA) model to articulate short- and long-term tobacco control goals. In 2013, BHWP conducted follow-up interviews by telephone with the participating agencies to determine outcomes for their system change goals.

## Results

Emergent themes were identified from baseline key informants (n = 17, Table 1): staff ambivalence, system challenges, insufficient resources, and tobacco’s lack of priority status, effective strategies, and creation of a movement. During the 4 regional trainings, attendees reported needing both county and organizational policies but noted lack of implementation and enforcement of tobacco-free policies due to inadequate time and resources. Legal guidance and health educational tools were requested. Attendees asserted that policies are possible but take time and multilevel buy-in. To increase buy-in from agencies, attendees proposed a top-down approach combined with a bottom-up approach, which required that all decision makers, staff, and clients be involved in tobacco-free policy development, implementation, and enforcement to ensure success. To aid policy implementation, attendees asked that health department champions be intermediaries between facilities and their decision-making boards.

**Table 1:** Common Themes at Baseline and Follow-up Interviews With California Public Health Agencies

Common Themes at Baseline Key Informant Interviews (n = 17 agencies)	No. of Agencies	Representative Comments
**Ambivalence of staff**		
Staff members smoke	6	“Hard to educate people about tobacco in 12-step programs because staff smoke.”
Harm reduction culture	3	“Challenges to get people to view tobacco as a drug like other drugs.”
Staff training	5	“Hard to change staff culture. They are stretched and do not have time to include tobacco and are not as experienced and knowledgeable [about tobacco cessation techniques as are cessation experts].”
		“Staff have competing priorities, demand hiring freezes, cuts to programs, layoffs, reduction in funding, and are trying to get their regular assignments done.”
Myths about safe treatment options	3	“Fear that the client will be in danger [of nicotine and medication interactions and/or a black market for tobacco products].”
		“Staff myth about safe treatment options, lack of education, and unwillingness to accept new culture.”
Cultural change is difficult	9	“Social environment and culture of clients.”
		“Universal experience of smoking.”
Address the myths	3	“Address the myths: eliminating smoking creates less violence in facilities, not more”; the myth that “state laws prohibit many kinds of smoke-free facilities.”
**Facility or system challenges**		
Enforcement is difficult	3	“Voluntary policies at county [agencies] are hard to enforce.”
		“Smoke-free policies are enforced by staff and signage, but are hard to enforce.”
Fear of losing business	7	“The recovery center that contracts with [the] county attempted to go smoke-free but they were ultimately afraid of losing business; clients do not want to come back.”
		“Terrible economy has created a very difficult environment for policy that impedes business.”
Enormity of the health system including mental health programs, alcohol and drug programs, public health programs	4	“It is a maze of programs — many different types of programs, wellness centers, private facilities; it is hard to get everyone on the same page.”
**Lack of resources**		
Lack of time and funding	7	“Budget cuts are priority; [the] county is not willing to work on policies at this time.”
Lack of training	3	“No access to training [for decision makers, clients, and staff]; no training means no information.”
Lack of tailored materials	13	“FAQs, easy to read, long-term benefits, key informant and poll surveys that others are on board and you can be successful.”
		“We need fact sheets and flow charts and education about how we can [create tobacco-free policies], how to be advocates for those who do not have a voice, voices for the community, we need information and the backstory that goes with it.”
**Tobacco is not a priority for staff**		
Tobacco is not a priority for staff	5	“Tobacco is not a priority when clients are recovering from other addictions.”
		“Tobacco is used as a stress management tool.”
**Effective strategies**		
Commitment to smoke-free policies	8	“County is working to prohibit smoking on all county property.” “Starting to get hospitals to go smoke-free and creating systems level changes in the county; once this happens, we will go after legislative policy, using models of change for momentum.”
Existing smoke-free partnerships	13	“Long-term relationships between the community, county programs and private facilities are] created by providing a lot of information and education — providing tools [to quit smoking], incentives, participation in our events.”
		“Need: trust, networking, relationship-building, connection with county departments, getting into the community and meeting people and finding common ground to establish link with BH [behavioral health] community.”
Tobacco champion to advance policy	9	“Need a community champion. We have a doctor that has made tobacco her life’s career. She trains all rotating residents on cessation services and hopes to integrate help from residents at community clinics.”
		“Need to reach out to decision makers but also need the support and buy in from the community.”
**Creating a movement**		
Need trainings	4	“Trainings need to be a call to action to motivate.”
		“When CEUs are provided, more behavioral health counselors will participate.”
County or state agency leadership	3	“We need a guiding agency to take the lead — like a statewide agency.”
		“County has a Building Better Health Initiative that is organizing and leveraging all programs to work together and focus on strategies.”
Community organizing	6	“Need community organizing around tobacco helping people getting from where they are to where they want to go.”
		“Make it a ‘wave of the future,’ working with urban developers.”
Inspirational stories	2	“Need a model for what projects have worked in other states or counties: inspirational stories.”
		“We need dialogue with stories of recovery.”
**Common themes at key informant interviews (n = 10 agencies)**		
Positive encouragement	5	“[Going smoke-free] was one of the best additions to our program and I believe has contributed to the continued sobriety [of] countless women.”
		“Many of the staff, alcohol and drug counselors, are ‘in recovery’ and are smokers. Cigarette smoking is accepted, and seen as a substance-based coping mechanism.”
		“No obstacles — as we have learned that getting pharmaceuticals is straightforward with public funds. We provide patches to all incoming clients who are smokers.”
Promoting cessation	2	“Cessation classes are regularly offered and actively promoted.”
		“We have an ongoing program to provide training to staff and patients alike.”
Tobacco-free policy adoption	2	"Yes, we did obtain leadership approval and we have been a tobacco-free facility since July 8th, 2013.”
		“We do not allow e-cigarettes at the facility, and I know this is an issue that has [been] shown to be of concern to other areas of the country as well.”

Most agencies were in early stages of policy implementation and planned to focus on staff and leadership awareness regarding the benefits of tobacco-free policies. Also, 15 county agencies requested and received additional technical assistance in the months following trainings.

Follow-up interviews with 3 county agencies and 7 nongovernment organizations showed that trainings identified specific achievable objectives (n = 10, Table 1, bottom section). Heightened resistance to policies was reported for addiction treatment centers and trauma agencies. Agencies accomplished about 50% of their goals, such as recruiting members for tobacco coalitions and educating decision makers. Progress is evidenced by growing community partnerships, increased tobacco-free policies (n = 2), and provision of cessation services (n = 2) (Table 1). County public health agencies in Sonoma, Monterey, Santa Cruz, Watsonville, Shasta, and San Diego partnered with behavioral health agencies to build coalitions. BHWP conducted trainings in these counties and others to implement train-the-trainer tobacco use cessation programs. We also found a decrease in the proportion of agencies in early stages of change (Table 2) because they had moved to a later stage. Organizations at the action and maintenance stages (Table 2) doubled from baseline to follow-up.

**Table 2:** Willingness of California Agencies^a^ to Adopt Tobacco-free Policies and System Change^b^

Stage of Change	Base (n = 45), %	Follow-up (n = 10), %
Precontemplation	24	24
Contemplation	36	29
Preparation	16	13
Action	4	11
Maintenance	7	11

a These 45 agencies represent unique government and nongovernment organizations that participated in 1 of the 4 trainings and submitted rapid improvement plans.

b Based on a convenience sample of agencies who responded to a request for key informant interviews. We assumed no change in stage for those agencies that did not respond during follow-up.

## Discussion

California has been effective in helping the behavioral health care system to address the health disparities experienced by smokers with behavioral health issues. Training participants expanded their knowledge and outlined concrete plans for transitioning to tobacco-free environments.

Obstacles to implementing policy still remain. Specifically, many agencies treating trauma and addictions continue to have concerns about going tobacco-free. In part, this reluctance reflects a long-held perspective that other issues are more important and that clients cannot or do not desire to quit smoking (2). Future training should acknowledge providers’ competing demands but also refute common misinformation that has reinforced inaction.

Building on the gains from statewide trainings, California is funding 4 additional county health departments to increase the number of smoke-free health care campuses (El Dorado, Lake, Mariposa, Placer), and 2 county health departments (Humboldt, Sonoma) are being funded to adopt or implement behavioral health care treatment programs.

CTCP held another series of regional trainings in 2014. The trainings have led to collaboration between the California Department of Public Health (CDPH) and the California Department of Health Care Services (DHCS). DHCS is currently implementing the Affordable Care Act (ACA) and the Medi-Cal expansion that supports comprehensive tobacco cessation programs for beneficiaries. The Medi-Cal expansion focuses on creating a plan of care that treats the whole person, including mental health and alcohol and drug services. CDPH and DHCS are working together to determine how best to integrate tobacco control activities into state Medicaid benefits and how to strengthen systemwide tobacco control policies.
